# Primary Cilia as a Biomarker in Mesenchymal Stem Cells Senescence: Influencing Osteoblastic Differentiation Potency Associated with Hedgehog Signaling Regulation

**DOI:** 10.1155/2021/8850114

**Published:** 2021-01-26

**Authors:** Su Fu, Chunlin Zhang, Xu Yan, Dongzhe Li, Yongkui Wang, Chao Dong, Zhengming Cao, Yongming Ning, Chenglong Shao, Tengyue Yang

**Affiliations:** Department of Orthopaedics, The First Affiliated Hospital of Zhengzhou University, China

## Abstract

Bone tissue engineering-based therapy for bone lesions requires the expansion of seeding cells, such as autologous mesenchymal stem cells (MSCs). A major obstacle to this process is the loss of the phenotype and differentiation capacity of MSCs subjected to passage. Recent studies have suggested that primary cilia, primordial organelles that transduce multiple signals, particularly hedgehog signals, play a role in senescence. Therefore, we explored the relationships among senescence, primary cilia, and hedgehog signaling in MSCs. Ageing of MSCs by expansion in vitro was accompanied by increased cell doubling time. The osteogenic capacity of aged MSCs at passage 4 was compromised compared to that of primary cells. P4 MSCs exhibited reductions in the frequency and length of primary cilia associated with decreased intensity of Arl13b staining on cilia. Senescence also resulted in downregulation of the expression of hedgehog components and CDKN2A. Suppression of ciliogenesis reduced the gene expression of both Gli1, a key molecule in the hedgehog signaling pathway and ALP, a marker of osteoblastic differentiation. This study demonstrated that the senescence of MSCs induced the loss of osteoblastic differentiation potency and inactivated hedgehog signaling associated with attenuated ciliogenesis, indicating that primary cilia play a mediating role in and are biomarkers of MSC senescence; thus, future antisenescence strategies involving manipulation of primary cilia could be developed.

## 1. Introduction

Bone tissue engineering-based therapies have been developed to treat some challenging bone lesions, e.g., skeletal defects [1, 2]. Due to the limited mass of bone substitutes, implantation always requires the expansion of seeding cells, such as autologous mesenchymal stem cells (MSCs), which are attractive candidates for use in regenerative treatment [3]. A major obstacle to this therapeutic strategy is therefore the loss of the phenotype and differentiation potential of senescent MSCs subjected to passage in vitro [4] or harvested from aged donors ex vivo [5] before clinical use. In detail, senescent MSCs exhibit decreased proliferative activity and differentiation potential and enlarged morphology and chromosomal instabilities [5]. For example, MSCs show a reduced capacity to differentiate into osteogenic lineages and downregulated the expression of alkaline phosphatase (ALP), collagen type 1 (Col I) [4], and RUNX2 [6] after serial passaging in vitro. The underlying mechanisms include the overexpression of the senescence-related gene CDKN2A/p16 [7], as well as p21, p53, and some cytokines and growth factors [8]. Several manipulation methods have been shown to influence MSC senescence. Genetic modification is possible, as knockdown of p16 restores the phenotype of senescent MSCs [7]. Alterations in culture conditions include the application of hydrogen gas [9], antioxidants [10], and mechanical stress [11]. The addition of some pharmaceuticals to cell culture, such as lysophosphatidic acid [12] and growth factors [12], also suppresses senescence. However, further research on the mechanism of MSC senescence is needed to identify novel targets and provide insight.

The hedgehog family consists of three protein ligands: Sonic hedgehog (SHH), Indian hedgehog (IHH), and Desert hedgehog (DHH). In the hedgehog signaling pathway, the receptor patched-1 (Ptch1) normally resides in the primary cilium. After binding to the ligand, Ptch1 is degraded and transported out of cilia, while the smoothened (Smo) protein is trafficked into the cilium and is activated, resulting in downstream effector activation. Mutants affecting the cilia expression and/or IFT result in disrupted hedgehog signaling [13]. A recent study showed that the IHH ligand prevents MSC senescence by inhibiting the gene expression of p16, p53, and SA-*β*-gal and promotes MSC osteogenesis [14]. Additionally, SHH was shown to stimulate the osteoblastic differentiation of MSCs [15], while a hedgehog antagonist was shown to delay fracture healing [16], suggesting the promoting role of hedgehog signaling in bone formation. Interestingly, the senescence marker CDKN2A is regulated by the hedgehog transcription factor Gli2 in primary cilia-dependent and cilia-independent manners [17]. Several studies have also indicated that senescence is controlled by hedgehog signaling associated with changes in primary cilia formation.

In this study, we mainly determined the influences of senescent MSCs on osteogenic differentiation, primary cilia expression, and hedgehog signaling activation. The changes in the frequency and length of primary cilia were in accordance with changes in senescence and hedgehog signaling, whereas inhibition of ciliogenesis suppressed the Gli1 and ALP expression. This evidence suggests that primary cilia play mediating roles in and are biomarkers of MSC senescence, providing new targets for the development of future antisenescence strategies.

## 2. Materials and Methods

### 2.1. MSC Culture

Rats were euthanised by cervical dislocation and then thoroughly cleaned by being submerged in alcohol for 2 minutes. To open the femur and tibia, a scalpel was used to cut the skin and muscles. Bone marrow was extracted by flushing using culture medium into a flask. The medium was prepared by supplementing L-Dulbecco's Modified Eagle's Medium (L-DMEM, Gibco, USA) with 10% foetal bovine serum (FBS, Gibco, USA), 100 IU/ml penicillin, and 100 *μ*g/ml streptomycin (PS). After 48 h, the medium was changed to remove the nonadherent cells. The attached cells were cultured in culture flasks and expanded until they reached 80–90% confluence. The cells isolated from the bone marrow tissue, pooled from three individual rats, were cultured and transferred to a 6-well plate as primary MSCs (defined as P0, seeding density of 10^5^/well) or passaged to P4 and then transferred to a 6-well plate (seeding density of 10^5^/well). Alternatively, MSCs were harvested from young or aged rats (1 month and 18 months old) via the protocol mentioned above. siRNA knockdown of IHH or IFT88 was performed using a standard protocol. MSCs were transfected with 100 nM siRNA targeting IHH or IFT88 or a scrambled sequence (negative control siRNA) (Guangzhou Bio Co., Ltd., China). The cultured cells were incubated at 37°C in 5% CO_2_.

Osteoblastic differentiation of MSCs was induced by treatment with 10 mM *β*-glycerophosphate, 100 nm dexamethasone, and 50 *μ*g/mL ascorbic acid, which is a well-established strategy [18]. We treated the cells for one week to explore the differentiation potency of MSCs because a highly mineralised layer of cultured MSCs forms after 2 weeks. This mineralization affects the ability to scrape the MSCs, resulting in an inhomogeneous RNA sample.

### 2.2. BMSC Identification

The P0 and P4 MSCs were analyzed for the expression of CD29, CD34, and CD44 via flow cytometry as previously described [19]. Before detection, the multiple Intelligences of the P0 MSC ability as adipogenic and chondrogenic differentiation was confirmed by our group. Then, approximately 5 × 10^4^ MSCs (P0 and P4) were transferred to tubes and then stained with phycoerythrin-conjugated anti-CD29, CD44, CD73, CD90, CD105, CD34, and CD44 for 30 min at 4°C. After washing with ice-cold PBS buffer, the expression of MSC markers was analyzed by flow cytometry (Beckman-Coulter Electronics, Hialeah, FL). The difference in the number of stained P0 MSCs and P4 MSCs was determined.

### 2.3. Detection of MSC Proliferation and Differentiation Capacity

To determine the proliferative activity of primary or passaged MSCs, cells were collected and stained with trypan blue to identify dead cells. The cell number was determined at the end of a 5-day culture period, and the cell doubling time was calculated based on a previous study [4]. To induce osteogenic differentiation, the MSC culture medium (DMEM +10%FBS + PS) was replaced with differentiation medium (*α*-MEM containing 10 mM *β*-glycerophosphate and 50 *μ*g/mL ascorbic acid) for one week. A longer time of induction (>2weeks) caused a formation of high mineralization layer preventing the scrape of MSCs and successful immunofluorescence labeling of primary cilia. ALP staining was utilised to evaluate the expression of ALP in MSCs cultured on coverslips with the Alkaline Phosphatase Staining Kit.

### 2.4. Immunocytochemical Staining

P0 and P4 MSCs were directly cultured on coverslips and were then fixed with 4% paraformaldehyde for 5 minutes. The cells were then washed 3 times in PBS and incubated for 5 min with 0.25% Triton-X/PBS to permeabilise the cells. Then, the cells were blocked with 5% goat serum/PBS for 1 hour. MSCs were incubated overnight with primary antibodies. Primary cilia were immuno-labeled using anti-acetylated *α*-tubulin (1 : 2000, T7451, Proteintech, USA) or anti-Arl13b antibody (1 : 2000, 17711-1-AP, Proteintech, USA). After incubation at 4°C overnight, the samples were washed 3 times in 0.1% BSA/PBS and then incubated with Alexa Fluor 488- and Alexa Fluor 633-conjugated secondary antibodies diluted 1 : 100 for 1 hour. The cells were counterstained with DAPI for 5 minutes to identify nuclei. Subsequently, the MSCs were washed three times in PBS. MSCs were also immune-immunofluorescent labeled with Ki-67 antigen (1 : 1000), thus calculating the percentage of positive cells. The slides were examined by confocal microscopy, and images were acquired using Image Manager software. Confocal z-stacks encompassing the entire depth of the cell (approximately 10 sections) were collected using a 0.5 *μ*m step size. Cilia length was quantified from the resulting maximum projection images using ImageJ, as in previous studies of chondrocyte cilia [20], which typically lie flat against the coverslip on the basal surface of the cell. The cilia area was measured as the number of pixels each cilia occupied on the maximum projection image, also used for. The number of cilia was determined from confocal reconstruction images in several random fields of view with the cilia prevalence (%) was calculated as the percentage of ciliated cells divided by whole cells.

### 2.5. Quantitative Polymerase Chain Reaction (qPCR)

To quantify the gene expression, MSCs were scraped and collected with TRIzol. cDNA was synthesised using a Quantitect reverse transcription kit (Invitrogen, USA). SYBR Green (Applied Biosystems, USA) was used for PCR. The amplification conditions were as follows: 95°C for 3 min followed by 40 cycles of 95°C for 15 s and 60°C for 30 s. Amplification was performed using the StepOnePlus™ Real-Time PCR System (Applied Biosystems, USA). ALP mRNA, Col l mRNA, RUNX2 mRNA, SMO mRNA, Ptch-1 mRNA, GLI 1 mRNA, and GLI 2 mRNA expression levels were normalized to GAPDH expression levels using the *Δ*Ct method. The primer sequences used in this study are listed in SI 1.

### 2.6. Western Blot Analysis

The cells were treated with RIPA lysis buffer to extract protein from whole cells. The proteins were subjected to 10% SDS-PAGE and transferred to a nitrocellulose membrane. Blotting was performed with primary antibodies against the osteogenic markers RUNX2 (sc-390351), ALP (sc-365765), and Col I (sc-59772), as well as CDKN2A (sc-1661) and IHH (sc-271101), which were all purchased from the Santa Cruz Biotechnology Company. GAPDH was used as the housekeeping protein. Enhanced chemiluminescence reagents were used to identify the bound antibodies. Finally, all bands were visualised and analyzed using ImageJ software.

### 2.7. Statistical Analysis

GraphPad Prism (GraphPad Prism 5.01, GraphPad Software, USA) was used to conduct all statistical analyses. Data from P0 and P4 MSCs are presented in the columns as the *means* ± *standard* *errors* of the mean (SEMs) and were mainly analyzed using Student's *t*-test. *p* < 0.05 was considered significant. In the histograms, the stars above the bars indicate significance in comparison to the corresponding group, while the # symbol indicates a difference from the control group without induction of osteogenic differentiation.

## 3. Results

### 3.1. Reduced Proliferative Activity and Preservation of the Surface Antigen Expression in Expanded MSCs

**Figure 1:**
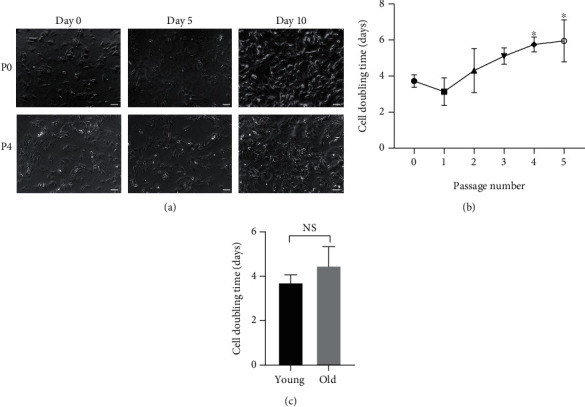
Changes in the morphology and proliferative activity of MSCs subjected to senescence induction. (a) Representation of morphological changes in primary MSCs (P0) and ageing P4 MSCs in vitro. Bright-field images of MSCs at P0 and P4 (captured on days 0, 5, and 10) showing that there were more proliferating P0 MSCs than P4 MSCs. *Scale* *bars* = 100 *μm*. (b) The reduction in proliferative capability was quantified by the MSC population doubling time, which showed a gradual increase with an increase in passage number. (c) However, primary MSCs from young animals and old animals did not show a difference in MSC doubling time. The numbers represent the mean values. *n* = 4; Student's *t*-test. ^∗^*p* < 0.05 versus the corresponding P0 MSCs cultured under the same expansion conditions.

MSCs were maintained in basic medium and then subjected to expansion. As shown in Figure 1(a), microscopy demonstrated that MSCs were spindle-shaped; on day 10, P0 MSCs were almost 100% confluent, but P4 MSCs were 80% confluent. The cell number doubling time (Figure 1(b)) showed a gradually increasing trend with increasing passage number. P4 and P5 MSCs exhibited a significantly longer doubling time than P0 MSCs (approximately 2-fold change), suggesting that expansion reduced proliferative capacity. However, there was no difference in cell doubling time between primary MSCs harvested from young animals and those harvested from old animals. This study, therefore, used primary (P0) and passaged (P4) MSCs to explore the mechanism of senescence.

The surface marker expression on primary and passaged MSCs was subsequently measured (SI 2) by flow cytometry to confirm the preservation of stem cells. P0 and P4 MSCs did not show different expressions of the specific MSC surface antigens CD29, CD44CD73 CD90 CD105 (very large population of positive cells, from 80% to 95%), or the specific HSC surface antigen CD34 and CD45 (from 1% to 4% of cells). The populations of P0 and P4 MSCs expressing these markers were similar.

### 3.2. Primary MSCs Exhibited better Osteogenic Potential than Aged P4 MSCs

**Figure 2:**
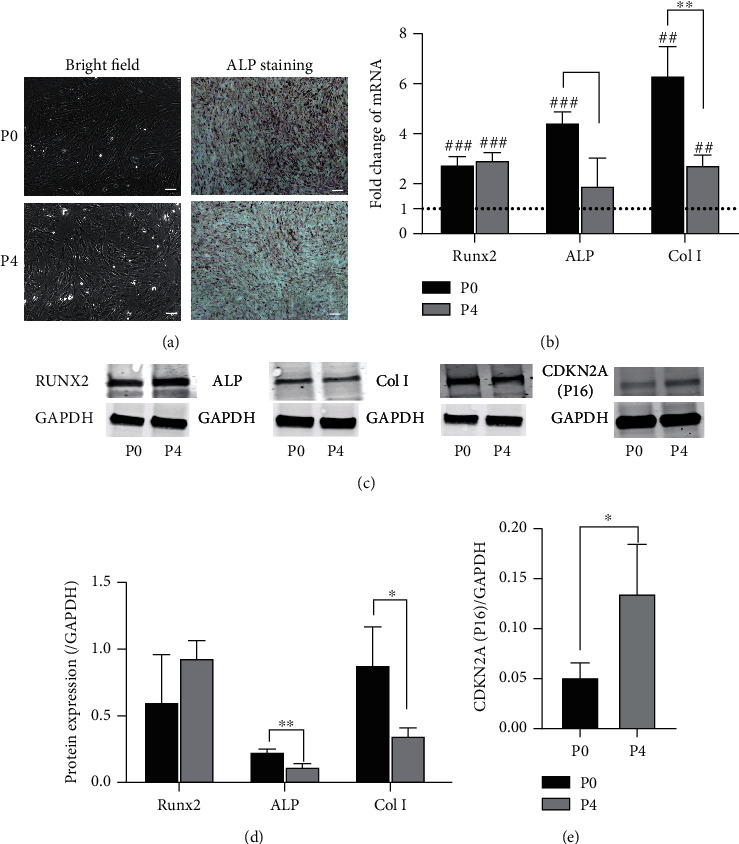
Osteogenic differentiation of P0 and P4 MSCs. Control or senescent cells were cultured with osteogenic differentiation medium for one week and subjected to ALP staining or detection of the gene and protein expression. (a) Representative bright-field images showing the changes in MSCs during differentiation induction and ALP staining, which was stronger in P0 MSCs than in P4 MSCs. *Scale* *bars* = 100 *μm*. (b) The expression of osteogenic genes (RUNX2, ALP, and Col I) was measured by qPCR with GAPDH as the reference gene. Student's *t*-test. ^∗^*p* < 0.05 versus the corresponding P0 MSCs cultured under the same differentiation conditions; ^#^*p* < 0.05 versus MSCs cultured in basic medium without induction of osteogenic differentiation. (c) The protein blots for RUNX2, ALP, Col I, and CDKN2A (p16) as well as (d) the quantification of the expression of these proteins relative to that of GAPDH showed that aged P4 MSCs exhibited decreased potency and increased expression of the senescence marker CDKN2A. All these results showed that the inhibition of osteogenic differentiation by expansion was associated with senescence. ^∗^*p* < 0.05 versus the corresponding P0 MSCs.

Next, we sought to identify the difference in osteogenic differentiation capacity between P0 and P4 MSCs. MSCs were cultivated with differentiation medium for one week, and both P0 and P4 MSCs were able to differentiate, as shown by bright-field images and ALP staining, suggesting the occurrence of osteoblastic differentiation (Figure 2(a)). Cell morphology of both P0 and P4 MSCs did not change significantly with a spindle-shape, arrangement in monolayer. MSCs gathered gradually to form a cell cluster with stronger ALP expression (greater colour reaction) was observed in P0 MSCs. Moreover, P0 MSCs expressed high levels of the genes ALP and Col, but not RUNX2, than P4 MSCs (Figure 2(b)). Similarly, the protein expression of ALP and Col 1 was upregulated in primary MSCs but not in RUNX2(Figures 2(c) and 2(d)). The protein expression of CDKN2A, which is a crucial factor for mediating senescence, was elevated in P4 MSCs (Figure 2(e)), confirming its association with senescence. These results indicated that senescent P4 MSCs possess a reduced level of osteogenic differentiation potency.

### 3.3. Reductions in Primary Cilia Number and Ciliary Arl13b Staining Intensity in Senescent MSCs

**Figure 3:**
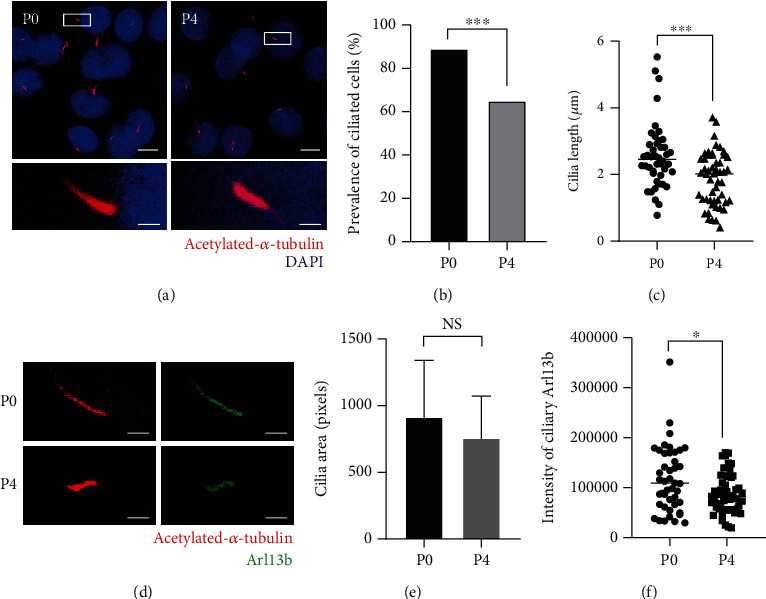
Expansion caused reductions in the number and length of primary cilia and Arl13b staining intensity on cilia in MSCs. Serum starvation was performed before primary cilia staining. P0 and P4 MSCs cultured on coverslips were analyzed by capturing confocal images of primary cilia. (a) Representative confocal microscopy maximum intensity projection images of MSCs labeled with acetylated *α*-tubulin (red) and DAPI (blue). The number of cilia (b) and cilia length (c) was determined. (d) Representative maximum intensity projection images of primary cilia immunofluorescence labeled with acetylated-*α*-tubulin (red) and Arl13b (green). The changes in the cilia area (e) and Arl13b staining intensity on cilia (f) are shown. The Mann–Whitney test. ^∗^*p* < 0.05 versus the corresponding P0 MSCs.

The associated changes in primary cilia were next explored via immunofluorescence staining of primary cilia for acetylated *α*-tubulin (red, Figure 3(a)) or Arl13b (green, Figure 3(d)). The confocal microscopy maximum intensity projection images of MSCs are shown in Figure 3(a), and they demonstrate a reduced number of primary cilia in senescent P4 MSCs, from over 80% ciliation in P0 MSCs to 60% in P4 MSCs. Ciliation and cilia length were calculated as described in our previous study [20]. In this study, the effect of serum starvation for 24 h before cilia measurement on MSC cilia was also explored (SI 3). Due to the inhibition of P4 MSC proliferation (SI 3A), serum starvation was used to arrest cells in the G0 phase. In the serum-maintained condition, the number of cilia was lower, but there was no difference between P0 and P4 MSCs. Conversely, in serum-starved cells, reductions in ciliation and cilia length were observed in P4 MSCs (SI 3B, C, and Figures 3(b) and 3(c)). We also measured the cilia expression in MSCs passaged 0-4 times (SI 3D, E) and found a gradual decrease in the number of cilia with expansion, consistent with the findings presented in Figures 1(b)and 2. It is likely that senescence is associated with changes in the number of primary cilia.

Due to the regulatory role of Arl13b in hedgehog signaling and its enrichment on cilia, we stained primary cilia for acetylated-*α*-tubulin to trace the cilia and Arl13b to evaluate its enrichment on cilia (Figure 3(d)). As expected, a decrease in the cilia area (Figures 3(e) and 3(a)) lower integrated intensity of Arl13b on cilia (Figure 3(f)) was observed in P4 MSCs, suggesting that there were changes in cilia and associated signaling.

### 3.4. Senescence in MSCs Was Accompanied by Inactivated Hedgehog Signaling

**Figure 4:**
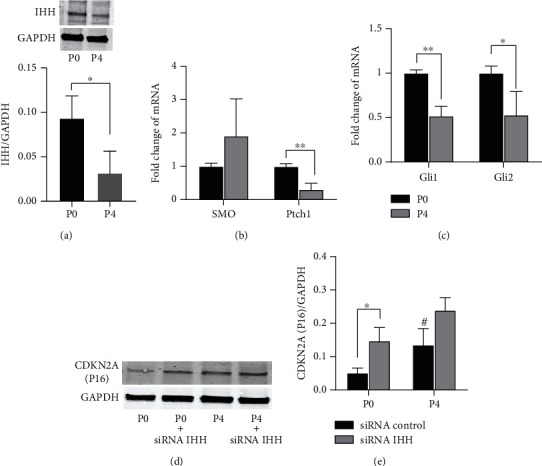
The associated inactivation of hedgehog signaling in senescent MSCs. (a) Blotting for IHH showing the decreased expression of IHH in P4 aged MSCs. PCR analysis indicated that expansion suppressed the gene transcription of Ptch-1 (b) and the transcription factors Gli1 and Gli2 (c). Suppression of the IHH expression by siRNA altered the CDKN2A expression. (d, e) Inhibition of the IHH increased CDKN2A protein expression and the difference between P0 and P4 MSCs were abolished. ^∗^*p* < 0.05, Student's t-test. ^#^*p* < 0.05 versus the corresponding P0 MSCs.

The involvement of hedgehog signaling in senescence was then explored by PCR and Western blot analysis of the expression of hedgehog components. The protein expression of the hedgehog ligand IHH was reduced in P4 MSCs (Figure 4(a)), suggesting that hedgehog signaling was inactivated in response to expansion. The mRNA expression levels of Ptch-1, Gli1, and Gli2 were also downregulated in aged P4 MSCs (Figures 4(b) and 4(c), approximately a 50% loss of gene expression). However, the transcriptional activity of SMO mRNA was not changed. Of these key hedgehog components enriched on cilia, Gli has been shown to regulate the CDKN2A expression [17]. Subsequently, siRNA IHH was used to abolish hedgehog signaling. IHH knock-out elevated the expression of CDKN2A in both P0 and P4 MSCs (Figures 4(d) and 4(e)), further suggesting the involvement of hedgehog signaling in MSC senescence.

### 3.5. Depletion of Primary Cilia by Mutation to IFT88 Leads to Downregulation of the Gene Expression of ALP and Gli1

**Figure 5:**
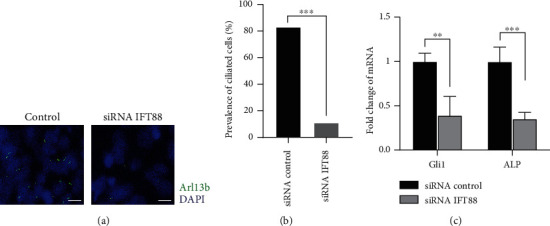
Depletion of primary cilia with a siRNA targeting IFT88 suppressed cilia formation and the gene expression of Gli1 and ALP. (a) Representative images showing the loss of primary cilia in P0 MSCs incubated with IFT88 siRNA. (b) The resultant decrease in ciliation was confirmed. (c) The gene transcriptional activities of Gli1 and ALP were compared, and it was found that loss of primary cilia suppressed hedgehog signaling and osteogenic differentiation. ^∗^*p* < 0.05, Student's *t*-test, versus MSCs treated with negative control siRNA.

To explore the role of primary cilia in MSC senescence, primary cilia were depleted by an siRNA targeting IFT88 (Figure 5(a)). The IFT88 gene-encoded intraflagellar transport protein IFT88 plays a central role in the primary cilia structure maintenance and function. Chloral hydrate was also added to ensure the removal of cilia. As a result, the loss of primary cilia was confirmed (Figure 5(a)), with a decrease in ciliation from over 80% to 10% (Figure 5(b)). Interestingly, the gene transcriptional activities of Gli1 and ALP were compared, and it was sound that the loss of primary cilia suppressed hedgehog signaling and osteogenic differentiation. These results indicated that primary cilia were required for hedgehog signaling-mediated regulation of MSC senescence via cilia-mediated key transduction factors such as Gli1.

## 4. Discussion

This study mainly demonstrated that the reduction in the osteogenic potency of MSCs subjected to expansion was accompanied by the loss of primary cilia and inactivation of hedgehog signaling, indicating the “primary cilia-hedgehog-CDKN2A” mechanism of MSC senescence. Our findings were consistent with previous studies in other cell types showing that the hedgehog ligand IHH promotes primary cilia formation in human mammary epithelial cells [17], suppresses senescence and CDKN2A expression in human MSCs [14], and induces downregulation of hedgehog signaling in passaged human fibroblasts [21]. A clear linkage was therefore proposed in which primary cilia were required for and/or resulted from hedgehog signaling, which regulated the senescence of MSCs, thus influencing osteogenic differentiation capacity.

We used cultured MSC expansion in vitro to monitor senescence-related changes, not MSCs from donors of different ages. Indeed, there is significant controversy regarding whether this kind of cell model represents ageing in vivo [22]. Expansion-induced replicative senescence has been widely used by a variety of researchers to identify senescence-associated genes [23, 24]. We did not observe a difference in the proliferation of MSCs from young and old donors, but a gradual loss of the proliferative activity was observed in passaged MSCs. Clear phenotypic changes in P4 MSCs were also shown, with cells exhibiting enlarged and rounded morphology, further confirming the utility of our expansion culture model. In terms of tissue origins, MSCs can be harvested from various tissues, such as adipose tissue. In this study, we used MSCs isolated from the bone marrow, which is considered the best-characterised source by far [3, 25]. MSCs have been shown to exhibit the decreased expression of stem cell-specific surface antigens [26]. We assessed several MSC markers and two HSC markers. As expected, MSCs exhibited a high proportion of cells expressing CD29, CD44, CD73, CD90, and CD105 but not CD34 and CD45. This maintenance of the MSC-marker expression was stable and previously seen in P10 [27] and even P80 [28].Consistent with that, our results suggested the stability of MSCs throughout the entirety of expansion.

Previously, we indicated the “structure-function” relationships between the regulation of mechanosignaling by primary cilia and inflammatory responses in articular chondrocytes [20, 29]. In this study, we found decreases in primary cilia length and number in MSCs after expansion, which is in accordance with previous studies in another cell type of chondrocytes [30] but not in fibroblasts [31]. Due to the nature of proliferative MSCs, with approximately 10-30% P0-P4 MSCs being positive for Ki67, serum starvation was used to arrest cells in the G0 phase, promoting ciliogenesis. This is also because the resorption of primary cilia during cycling leads to a misestimation when comparing primary cilia number and length. Based on the calculation, we also found an elevated Arl13b staining intensity on cilia in P0 cells compared with aged P4 cells, which demonstrates the probable linkage between cilia morphology and hedgehog signaling. Arl13b is a regulatory GTPase highly enriched in cilia that regulates hedgehog signaling [32]; in this study, the changes in the Arl13b expression on cilia may have had an impact on the transduction of hedgehog components that regulate senescence. However, a recent study showed that Arl13b also functions outside of primary cilia [33], which is undesirable but indicates the complexity of the regulatory mechanism within cilia. We also found that in senescent P4 MSCs, suppression of the IHH, Ptch-1, Gli1, and Gli2 expression was associated with a reduction in the CDKN2A expression. This reduced activation of IHH has been previously reported in senescent MSCs [14], which in turn affects the downstream expression of active Gli1 and Gli2. The CDKN2A promoter region has been revealed to have a Gli2 strong binding site [17], indicating the direct regulation of Gli2 by CDKN2A. Previous studies have shown that the depletion of cilia resulting from a mutation in IFT88 leads to decreased bone density [34] and inactivation of hedgehog signaling [35]. Similarly, in this study, loss of primary cilia suppressed the gene transcription of both Gli1 and ALP in P0 MSCs, suggesting the mediating function of primary cilia.

Other factors that affected MSC senescence also caused changes in primary cilia, further highlighting their involvement. For example, depletion of CDKN2A or exogenous IHH protein promotes an increase in the number of primary cilia formed in epithelial cells [17]. Mechanical loading in the form of strain or compression also causes cilia shortening and reduces hedgehog signaling [36, 37]. Oxygen tension mediates senescence [38] while regulating primary cilia disassembly [39]. These factors may change the morphology of MSC primary cilia, probably affecting the trafficking of key hedgehog components, such as SMO, Arl13b, and Ptch1, to cilia and thus regulating hedgehog signaling and senescence. Further studies are needed to elucidate these potential regulators.

In conclusion, we mainly identified the mechanism by which primary cilia mediate hedgehog signaling to regulate MSC senescence and therefore influence osteogenic potency, which is associated with changes in primary cilia number and length. As a result, primary cilia could be biomarkers of senescence or futures targets for the manipulation of MSC senescence.

## Data Availability

The data used to support the findings of this study are included within the article.

## Supplementary Materials

Supplementary Materials Supplementary Table 1 (SI 1) listed the primer sequences of ALP, GAPDH, RUNX2, Col I, Ptch1, Smo, and Gli1 for qPCR used in this study. Supplementary Figure 2 (SI 2) accessed the stemness of P0 and P4 MSCs used in this present study by detecting the expressions of surface antigen markers via flow cytometry. Supplementary Table 3 (SI 3) showed the additional information on primary cilia prevalence and length data in primary or passaged MSCs.
