# Supplementary material for: Primary Cilia as a Biomarker in Mesenchymal Stem Cells Senescence: Influencing Osteoblastic Differentiation Potency Associated with Hedgehog Signaling Regulation
Source: Stem Cells Int. 2021 Jan 26;2021:8850114. doi: 10.1155/2021/8850114 (PMC7857927; doi:10.1155/2021/8850114)
Supplement: Supplementary Materials — Supplementary Table 1 (SI 1) listed the primer sequences of ALP, GAPDH, RUNX2, Col I, Ptch1, Smo, and Gli1 for qPCR used in this study. Supplementary Figure 2 (SI 2) accessed the stemness of P0 and P4 MSCs used in this present study by detecting the expressions of surface antigen markers via flow cytometry. Supplementary Table 3 (SI 3) showed the additional information on primary cilia prevalence and length data in primary or passaged MSCs. [file 8850114.f1.docx]

**Supplementary materials**

**Primary cilia as biomarkers of mesenchymal stem cell senescence: influence on osteoblastic differentiation potency associated with Hedgehog signalling regulation**

Su Fu (MD)^1^,* Chunlin Zhang (MD)^1^, Xu Yan (MD)^1^, Dongzhe Li (MD)^1^, Yongkui Wang (MD)^1^, Chao Dong (MD)^1^, Zhengming Cao (MD)^1^, Yongming Ning (MD)^1^, Chenglong Shao (MD)^1^, Tengyue Yang (MD)^1^

^1^Department of orthopaedics, the first affiliated hospital of Zhengzhou University, China

SI 1. The primer sequences used in this study

| **Gene** | **Sequence** |
| --- | --- |
| ALP | F: AACCCAGACACAAGCATTCC  R: GCCTTTGAGGTTTTTGGTCA |
| GAPDH | F: TGAGGTGACCGCATCTTCTTG  R: TGGTAACCAGGCGTCCGATA |
| RUNX2 | F: GAGCTACGAAATGCCTCTGC  R: GGACCGTCCACTGTCACTTT |
| Col I | F: TGGTCCTCAAGGTTTCCAAG  R: TTACCAGCTTCCCCATCATC |
| Ptch1 | F: TGGTCACACGAACAATGG  R: TGAACTGGGCAGCTATGAAGTC |
| Smo | F: AGTTACATCGCAGCCTTC  R: CACACTACTCCAGCCATC |
| Gli1 | F: TGCTGACACTCTGGGATA  R: CAGGGCCATAGTTGGTT |


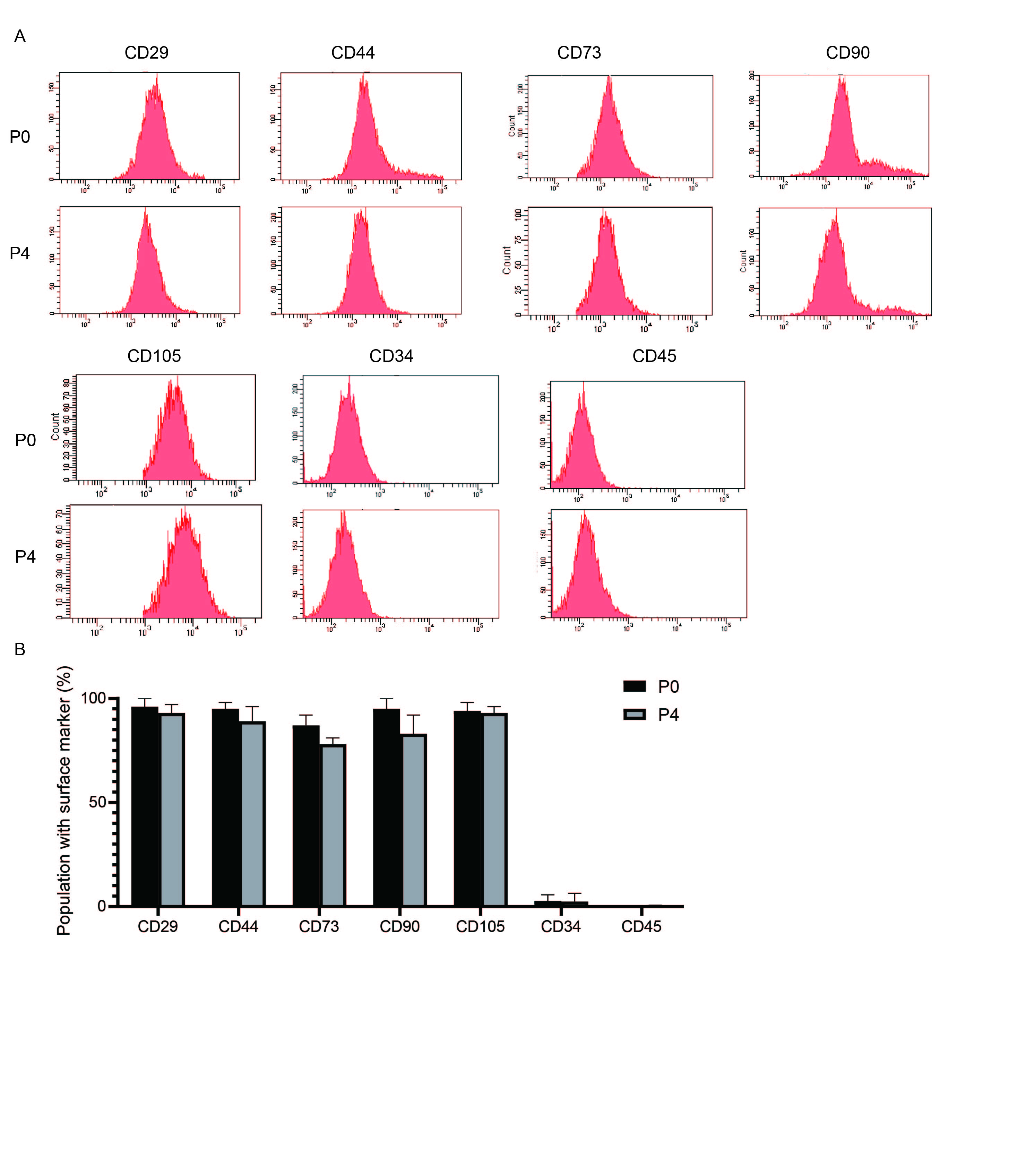


SI 2. Surface marker expression of P0 and P4 MSCs. The expressions of surface antigen markers were analyzed by flow cytometry. (A) The distribution of MSCs with the expression of specific MSC positive CD29/CD44/CD73/CD90/CD105, and negative CD34/CD45. n=3. (B) The population of MSCs with positive markers indicated the preservation of stem cell characters. Student’s t-test. *p < 0.05, versus the corresponding P0-MSCs cultured under the same expansion condition.


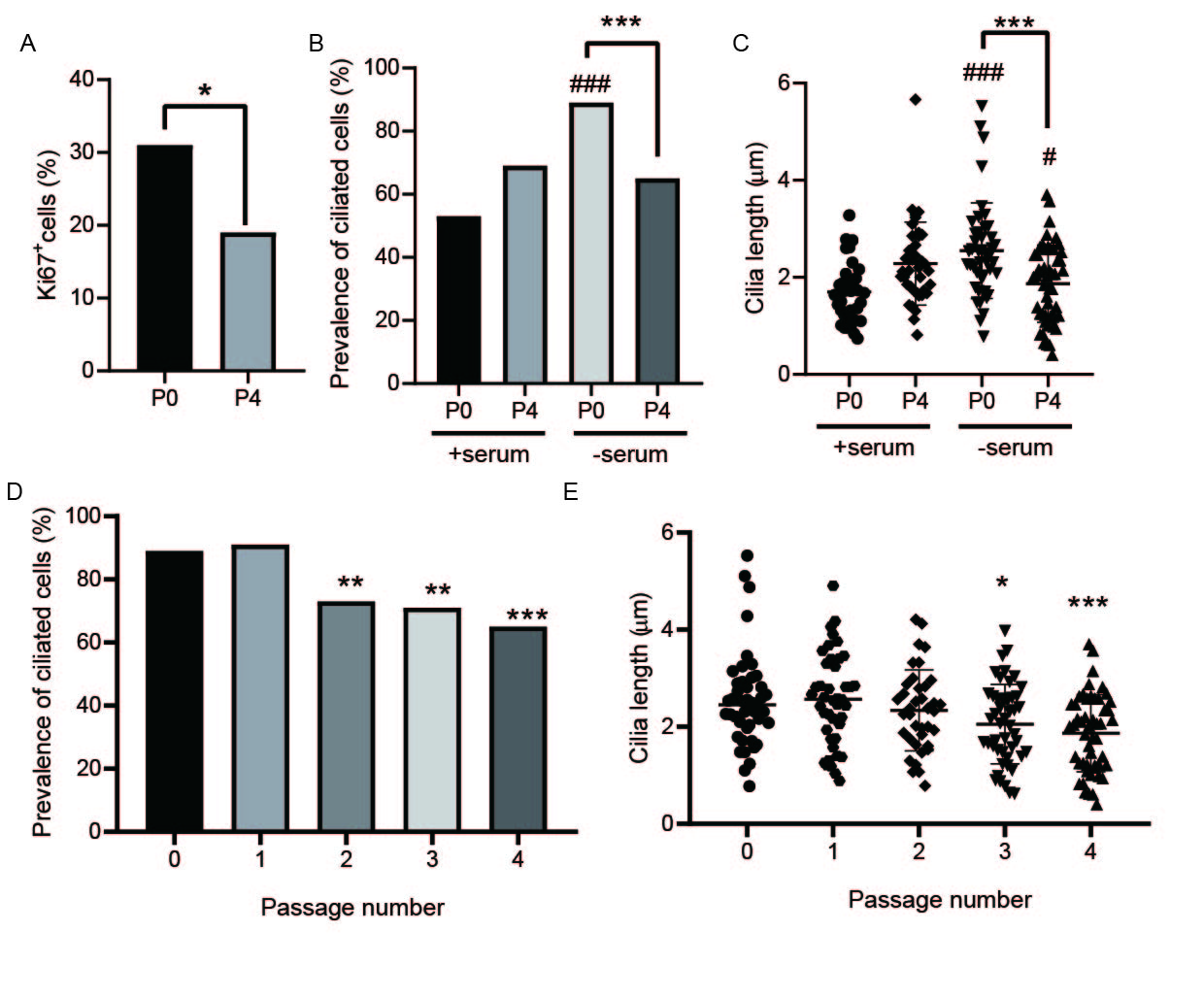


SI 3. Additional information on primary cilia expression in primary or passaged MSCs. (A) Percentage of Ki67 positive cells showed a decrease in proliferation of MSCs, which was consistent with Figure 1B but leads to an underestimation on the evaluation of primary cilia expression because cilia would disassembly during cycling. (B) The difference in cilia prevalence between P0 and P4 MSCs was only significant in serum-starved culture. Serum deprivation increased the prevalence of primary cilia (#).Mann-Whitney test.*p < 0.05, versus the corresponding P0 MSCs. (C) Similar to primary cilia frequency data, serum starvation was able to increase cilia length. P4 MSCs exhibited shorter primary cilia comparing to P0. (D) the ciliation and (E) cilia length in passaged MSCs, indicating a gradual decrease in cilia expression during expansion. Mann-Whitney test.*p < 0.05, versus the corresponding P0 MSCs.
